# Hotspots of Predictability: Identifying Regions of High Precipitation Predictability at Seasonal Timescales From Limited Time Series Observations

**DOI:** 10.1029/2021WR031302

**Published:** 2022-05-24

**Authors:** Antonios Mamalakis, Amir AghaKouchak, James T. Randerson, Efi Foufoula‐Georgiou

**Affiliations:** ^1^ Department of Civil and Environmental Engineering University of California Irvine CA USA; ^2^ Department of Atmospheric Science Colorado State University Fort Collins CO USA; ^3^ Department of Earth System Science University of California Irvine CA USA

**Keywords:** predictability, seasonal precipitation, extremes, drought, copula models, sea surface temperatures (SSTs), El Niño‐southern oscillation, ocean indices

## Abstract

Precipitation prediction at seasonal timescales is important for planning and management of water resources as well as preparedness for hazards such as floods, droughts and wildfires. Quantifying predictability is quite challenging as a consequence of a large number of potential drivers, varying antecedent conditions, and small sample size of high‐quality observations available at seasonal timescales, that in turn, increases prediction uncertainty and the risk of model overfitting. Here, we introduce a generalized probabilistic framework to account for these issues and assess predictability under uncertainty. We focus on prediction of winter (Nov–Mar) precipitation across the contiguous United States, using sea surface temperature‐derived indices (averaged in Aug–Oct) as predictors. In our analysis we identify “predictability hotspots,” which we define as regions where precipitation is inherently more predictable. Our framework estimates the entire predictive distribution of precipitation using copulas and quantifies prediction uncertainties, while employing principal component analysis for dimensionality reduction and a cross validation technique to avoid overfitting. We also evaluate how predictability changes across different quantiles of the precipitation distribution (dry, normal, wet amounts) using a multi‐category 3 × 3 contingency table. Our results indicate that well‐defined predictability hotspots occur in the Southwest and Southeast. Moreover, extreme dry and wet conditions are shown to be relatively more predictable compared to normal conditions. Our study may help with water resources management in several subregions of the United States and can be used to assess the fidelity of earth system models in successfully representing teleconnections and predictability.

## Key Points

- We present a rigorous probabilistic framework and novel statistical metrics to assess the predictability of seasonal precipitation totals
- Hotspots of winter precipitation predictability occur in the Southwest and Southeast of contiguous United States (CONUS)
- Sea Surface Temperature (SST) information may help with predicting dry or wet conditions in some areas, while normal conditions are difficult to predict

## 1 Introduction

It is well established that precipitation predictability on seasonal timescales draws upon information about ocean dynamics, which is considered as the principal forcing variable of the atmospheric circulation that ultimately drives regional hydroclimate (Charney & Shukla, 1981; DelSole & Banerjee, 2017; Goddard et al., 2001; Hao et al., 2018; Khan et al., 2017; Palmer & Anderson, 1994; Sankarasubramanian et al., 2009; Zimmerman et al., 2016). Sea surface temperatures (SSTs), which provide a partial constraint on ocean circulation, are typically used for prediction either with deterministic models (i.e., climate model simulations, also broadly known as dynamical models) or statistical models that aim to exploit physically and historically established teleconnections of precipitation with large‐scale modes of climate variability (e.g., El Niño‐Southern Oscillation, ENSO; see Dai, 2013; Enfield et al., 2001; Hao et al., 2018; Lindsey, 2016; McCabe & Dettinger, 1999; McCabe et al., 2004; Newman et al., 2016; Redmond & Koch, 1991; Schonher & Nicholson, 1989). However, both approaches face important limitations, and reliable prediction of seasonal precipitation remains a challenge (National Academics of Sciences, 2016; Wang et al., 2009). Limits to predictive skill of dynamical models arise from epistemic uncertainties (like incomplete knowledge of the underlying processes, uncertain initial conditions, uncertainties in model physics and limits on computational resources that place constraints on the grid resolution used in operational systems; Becker et al., 2014; Chang et al., 2000; Mamalakis et al., 2017), as well as aleatoric uncertainties due to the chaotic nature of the system. Similarly, empirical statistical models exhibit limited predictive skill, arising primarily from the complex nature of the relationship between large scale modes and regional hydroclimate, and the limited sample size that is available to build robust predictive models.

In the United States, expectations have been set in the recent report by the National Academies of Sciences, Engineering and Medicine (National Academics of Sciences, 2016), that seasonal predictions would, in 10 years, be used like weather forecasts are today. Under this premise and given the still challenging nature of the problem, it is important to recognize that in the Earth system there are periods and regions of inherently higher and lower climate predictability. For example, the so‐called “forecasts of opportunity” (Mariotti et al., 2020; Mayer & Barnes, 2021) correspond to time windows of enhanced predictability for the climate system, which arise from specific climate modes like ENSO being active. When one of these climate modes is active, it can lead to persistent and consistent anomalous responses in the atmospheric circulation (e.g., quasi‐stationary Rossby waves) that drive regional hydroclimate, thus, enhancing climate predictability. As recommended by National Academics of Sciences, (2016), broader acknowledgment and communication that the predictability of the climate system varies temporally and spatially would more adequately address user community expectations for skillful prediction and actionable information (Mariotti et al., 2020).

Here, we are interested in the spatial variation of predictability and recognize that there exist regions with inherently higher levels of predictability, that is, “hotspots of predictability” that are often surrounded by regions where predictive skill is considerably lower. Physically, hotspots of precipitation predictability are regions where the forcing signal of large‐scale climate modes (like ENSO) to precipitation is robust and is not overwhelmed by chaotic internal variability. In these regions the forcing signal from climate indices has a high signal‐to‐noise ratio in explaining past precipitation observations (Wang et al., 2017). Quantitatively, we herein define hotspots of predictability as regions where the prediction skill derived from large‐scale climate indices is statistically distinguishable from a climatology‐based prediction (at a *p* < 0.05 level), when evaluated considering prediction uncertainties and using an out‐of‐sample test data set. In contrast, surrounding regions where climate indices do not yield statistically significant predictions for out‐of‐sample test data are defined as low (or zero) predictability regions.

Under this setting, we revisit the problem of seasonal precipitation prediction across the contiguous United States (CONUS) and aim to gain insight into mechanisms structuring hotspots of precipitation predictability. Specifically, we focus on the winter months (Nov–Mar) – a time of the year with elevated fire risk for southeastern states (Li et al., 2020; Nowell et al., 2018) and when most precipitation occurs in western states – and attempt to answer the following questions: (a) In which CONUS regions is winter precipitation inherently more predictable? (b) How is predictability distributed across different classes of precipitation totals (dry, normal, wet)? (c) Are dry and wet extremes more or less predictable than normal conditions? (d) What are the sources of predictability, that is, the large‐scale climate modes that contribute to predictability? Although prediction of precipitation across different CONUS regions has been explored in the past (Gibson et al., 2021; Liu et al., 2018; Madadgar et al., 2016; Mamalakis et al., 2018; McCabe & Dettinger, 1999; Pan et al., 2019; Schonher & Nicholson, 1989; Stevens et al., 2021; Zarekarizi et al., 2018; Zhang et al., 2018; Zimmerman et al., 2016), fewer studies have approached the problem focusing on the entire CONUS (Devineni & Sankarasubramanian, 2010a, 2010b; Gershunov & Cayan, 2003; Schubert et al., 2016), and to the best of our knowledge, our study is the first to explore hotspots of predictability and to systematically address the four questions described above. Results from our analysis may improve water resources management at a federal level, by integrating the knowledge of how predictability is distributed spatially across the US for both dry and wet precipitation extremes.

In our approach, we assess seasonal predictability by recognizing that one of the key problems in this setting is the limited sample size of high‐quality time series observations. The small sample size cautions against overinterpreting predictability when prediction uncertainty has not been quantitatively assessed and calls for also addressing the high risk of overfitting (DelSole & Banerjee, 2017; Ham et al., 2019; Stevens et al., 2021). So far, no generalized framework of how to assess predictability under this setting exists in the literature. Indeed, to avoid overfitting, previous studies often have used simplified models that rely on a small number of predictors and thus may not leverage the full structure of global SST information (L’Heureux et al., 2015; Liu & Negrón Juárez, 2001; Lloyd‐Hughes & Saunders, 2002; Manatsa et al., 2017; Wu et al., 2009). Also, most of these studies have not systematically evaluated prediction uncertainty, limiting assessment of predictability from a signal versus noise perspective and the use of the findings in an operational setting.

The probabilistic prediction framework we present integrates several complementary elements. First, we use copula modelling to enable prediction across the full distribution of precipitation, including dry and wet tails, and to provide quantitative estimates of uncertainty. Second, we use principal component analysis as a dimensionality reduction technique to provide a compressed representation of the climate index predictors across multiple ocean basins and avoid overfitting. Third, we use cross validation to avoid overestimating predictive skill and ensure a rigorous uncertainty assessment. In our analysis, we predict the entire precipitation distribution function for each year in the last five decades over CONUS at a 1° × 1° spatial resolution. To answer questions (b) and (c) above, we make use of a multi‐category 3 × 3 contingency table (an extension of the typical 2 × 2 table) that is not commonly used in seasonal prediction settings, but (as we show) can offer insights about predictability across different precipitation classes. For the expanded contingency table, we also derive a modified version of the critical success index (CSI; Schaefer, 1990) that accounts for the uncertainty in the prediction, and we assess predictability for three separate classes of precipitation totals (dry, normal, wet), which are defined based on a range of probability levels. The asymptotic, theoretical values of the modified CSI statistic under the null hypothesis of no predictive skill are presented, and their finite‐sample distributions are obtained using Monte Carlo simulation. Finally, to gain physical insight on the sources of predictability, we investigate which climate indices are the most important predictors of precipitation in predictability hotspots.

The structure of the paper is the following. In Section 2, we describe the spatial domain and period of our analysis, the datasets we used to assess predictability, the copula‐based predictive model, the multi‐category 3×3 contingency table, and the modified CSI. In Section 3, we present the results of our analysis across CONUS and identify hotspots of winter precipitation predictability. In Section 4, we provide context for our main findings and identify directions for future research.

## 2 Prediction Setting and Methodology

### 2.1 Overview and Data Sets

The predictability of winter (Nov–Mar) precipitation totals across the contiguous US (CONUS) is of high practical and scientific interest. Nov–Mar is when most precipitation occurs for many states in the West, while it coincides with the fire season (or its onset) for many states in the Southeast (Li et al., 2020; Nowell et al., 2018). Thus, early and accurate prediction of precipitation is directly related to the ability of practitioners to efficiently manage water resources and mitigate fire risk, with important consequences for agricultural planning, food security and air quality. Indeed, limited prediction skill has led to considerable economic damage in the past (on the order of billions of US dollars), with the recent multi‐year California drought in 2012–2016 serving as a prominent example (AghaKouchak et al., 2015; Howitt et al., 2014, 2015; Medellín‐Azuara et al., 2016).

**Figure 1:**
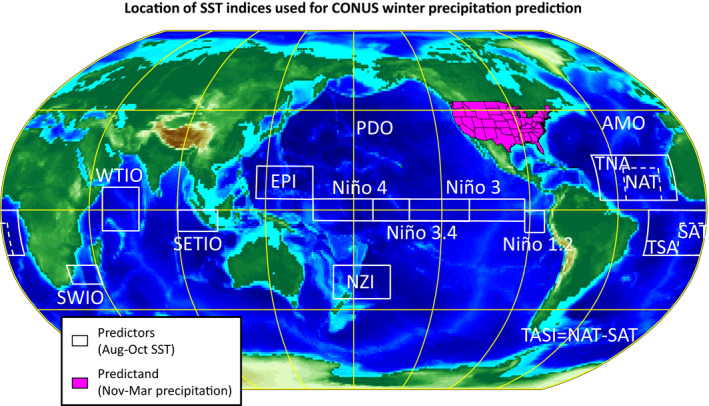
The 14 Sea Surface Temperature (SST) predictors (white boxes), averaged in Aug–Oct, used in this study to predict contiguous United States (CONUS) precipitation in the following Nov–Mar (magenta region) from 1967–1968 to 2016–2017. More information about the SST indices is provided at NOAA's State of the Ocean website (http://stateoftheocean.osmc.noaa.gov/) and in Chen et al. (2016).

To assess predictive skill of the CONUS precipitation, we used 14 SST indices (see white boxes in Figure 1) averaged over Aug–Oct to predict CONUS precipitation totals in the following Nov–Mar (i.e., there was no temporal overlap between the predictors and the predictands). We focused on the 50‐year period from 1967–1968 to 2016–2017 to build and test our predictive models. We avoided considering earlier years in the record because of the low spatial and temporal resolution of the early SST observations (see Figure 3 in Deser et al., 2010), and because the 1970s is roughly the time that many studies have pinpointed as the start of major changes in climate teleconnections (Johnson et al., 2019; Mamalakis et al., 2018, 2019; Swain et al., 2016; Wang et al., 2014). Both the precipitation and SST time series were linearly detrended before they were used in the analysis, so that long term trends do not impact our assessment.

**Figure 2:**
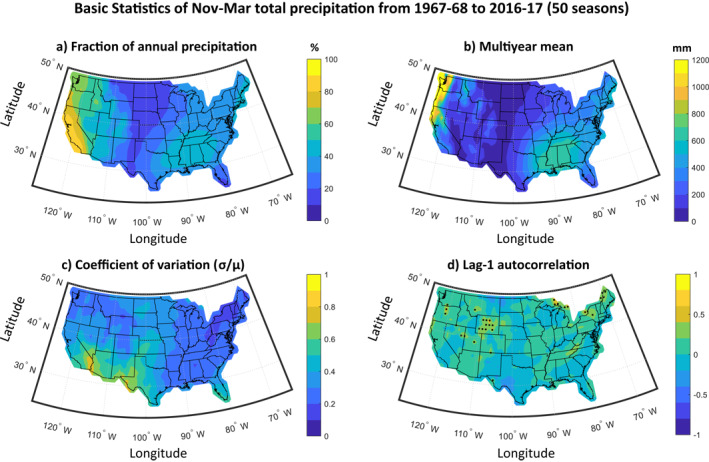
Summary statistics of Nov–Mar precipitation over contiguous United States (CONUS), during the years from 1967–1968 to 2016–2017 (50 seasons). (a) Fraction of annual precipitation that occurs during the Nov–Mar period. (b) Multiyear average of Nov–Mar precipitation. (c) Coefficient of variation of Nov–Mar precipitation (defined as the standard deviation divided by the multiyear average). (d) Temporal autocorrelation of the precipitation series for a 1 year lag. Stippling indicates where there is statistically significant autocorrelation (*p* < 0.05).

For our analysis, we used gridded observations at a 1° × 1° resolution of Nov–Mar precipitation amount (upscaled using spatial averaging from the original 0.25° × 0.25° resolution), which are freely available from the NOAA Climate Prediction Center (CPC) at https://psl.noaa.gov/data/gridded/data.unified.daily.conus.html (Chen et al., 2008). In Figure 2, we show several basic statistics of precipitation across CONUS, during the period of the analysis. For western states, Nov–Mar is when the most precipitation occurs (see Figure 2a), while for many eastern and southeastern states, this season accounts for less than 50% of the total annual precipitation and coincides with the regional fire season (or its onset) (Li et al., 2020; Nowell et al., 2018). This means that for most of the CONUS, predicting Nov‐Mar precipitation may aid with effective water planning and/or mitigation of fire risk. On average, the highest precipitation during Nov–Mar occurs over the western states, often exceeding 1,000 mm per season, while the highest year‐to‐year variability is shown over the southwestern US (Figures 2b and 2c; also see Dettinger et al., 2011; Dettinger & Cayan, 2014). Our results also show that there is no statistically significant temporal autocorrelation in the precipitation series for the majority of CONUS grid points (see Figure 2d). This implies that there is no significant year‐to‐year dependence, and practically, there is no predictive potential in using the past values of precipitation to predict in the next year.

**Figure 3:**
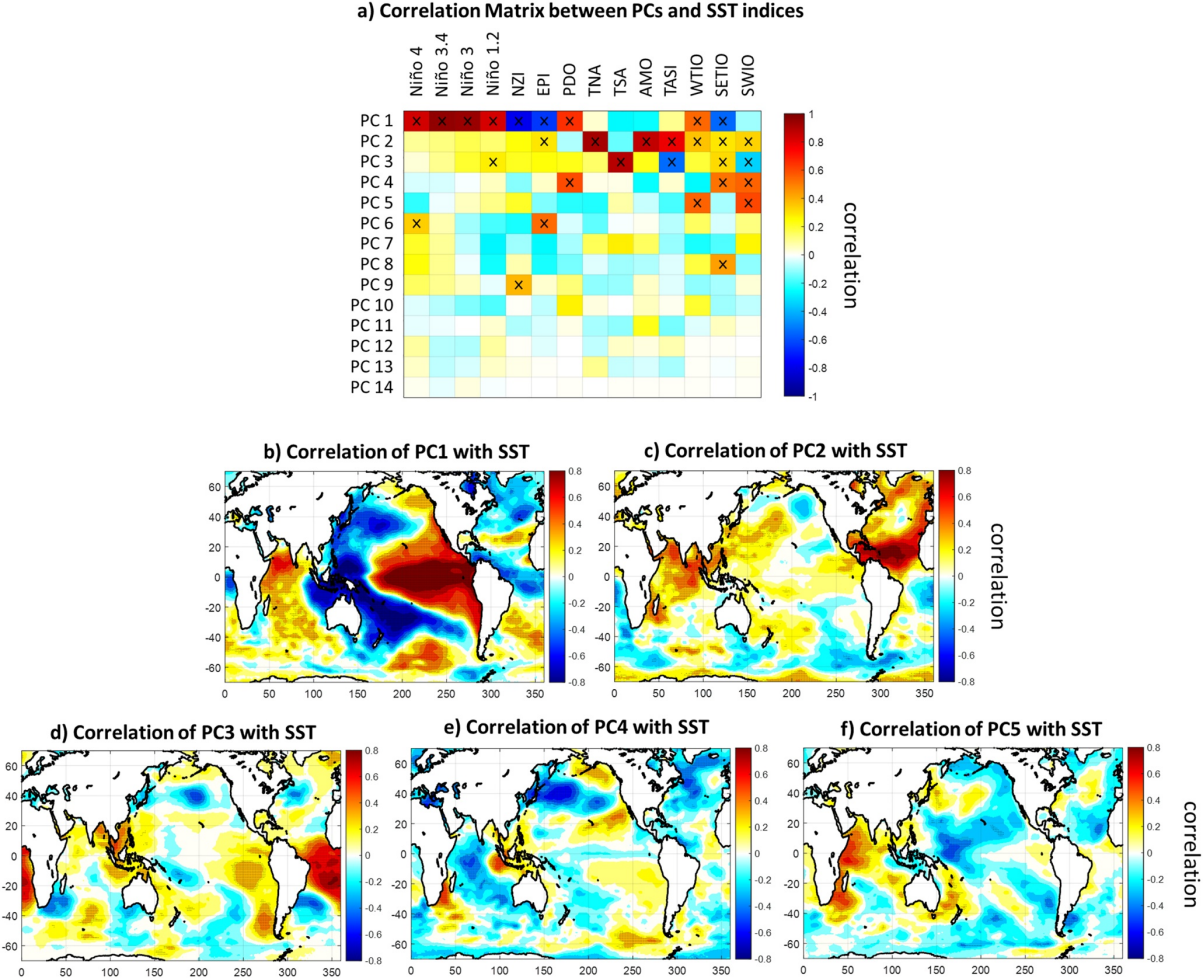
Spatial patterns of the principal components (PCs) of the sea surface temperature (SST) indices. (a) Correlation matrix of the PCs with the SST indices. (b)‐(f) Correlation maps of the first five PCs with SST. SST series in each grid point corresponds to the average SST in the Aug‐Oct season. All series are detrended before the analysis. Symbols “x” in panel (a) and stippling in other panels indicate statistically significant correlation (*p* < 0.05).

As predictor variables, we examined various SST indices in three ocean basins (Figure 1). Specifically, we examined Niño 4, Niño 3.4, Niño 3, and Niño 1.2 indices from the Pacific Ocean, TNA, TSA, AMO, and TASI indices from the Atlantic Ocean, and WTIO, SETIO, and SWIO indices from Indian Ocean. More information about these indices is provided at NOAA's State of the Ocean website (http://stateoftheocean.osmc.noaa.gov/) and in Chen et al. (2016). We also considered two recently introduced Pacific indices, that is, the New Zealand Index (NZI; 40°S–25°S and 170°E–200°E) and the East of the Philippines Index (EPI; 5°N–20°N and 130°E–160°E), which have been recently shown to exhibit strong statistical relationship with precipitation over the southwestern US (Mamalakis et al., 2018, 2019). The series of all above indices were constructed using monthly SSTs on a 1° × 1° grid that were obtained from version 2 of the Centennial In Situ Observation‐Based Estimates (COBE SST2; https://www.esrl.noaa.gov/psd/data/gridded/data.cobe2.html; Hirahara et al., 2014). Additionally, a monthly time series of PDO was directly obtained from the NOAA website https://www.esrl.noaa.gov/psd/data/climateindices/list. For our analysis, we used the series of all 14 SST indices (temporally averaged during Aug–Oct), considering them both individually and combined, while we also built predictive models using their principal components (PCs). This allowed us to retain most of the SST variability (e.g., the first and second PCs capture 38.5% and 19.7% of the variance of the 14 indices, respectively, i.e., yielding a total of nearly 60%), without considerably increasing the complexity of the predictive model, and thus, avoid overfitting (i.e., when thinking in terms of a linear regression model, using the first two PCs required the estimation of only two regression coefficients, rather than the much larger number of parameters required to access the same level of variance from the set of uncompressed ocean indices). In Figure 3, we present the spatial patterns of the first five PCs. This analysis revealed that each PC originates primarily from a different ocean basin, which helped us to draw physical insight about the different sources of predictability. The first PC characterizes SST variability in the Pacific (and resembles an ENSO pattern), the second and third PCs characterize variability in the Atlantic (resembling the Atlantic meridional and equatorial modes, respectively), while the fourth and fifth PCs capture considerable SST variability in the Indian Ocean (see an Indian Ocean dipole pattern in PC4). Higher order PCs correspond to only 10% of the total SST variability cumulatively, and so they were not further analyzed in our study.

### 2.2 Predictive Model to Assess Predictability

**Figure 4:**
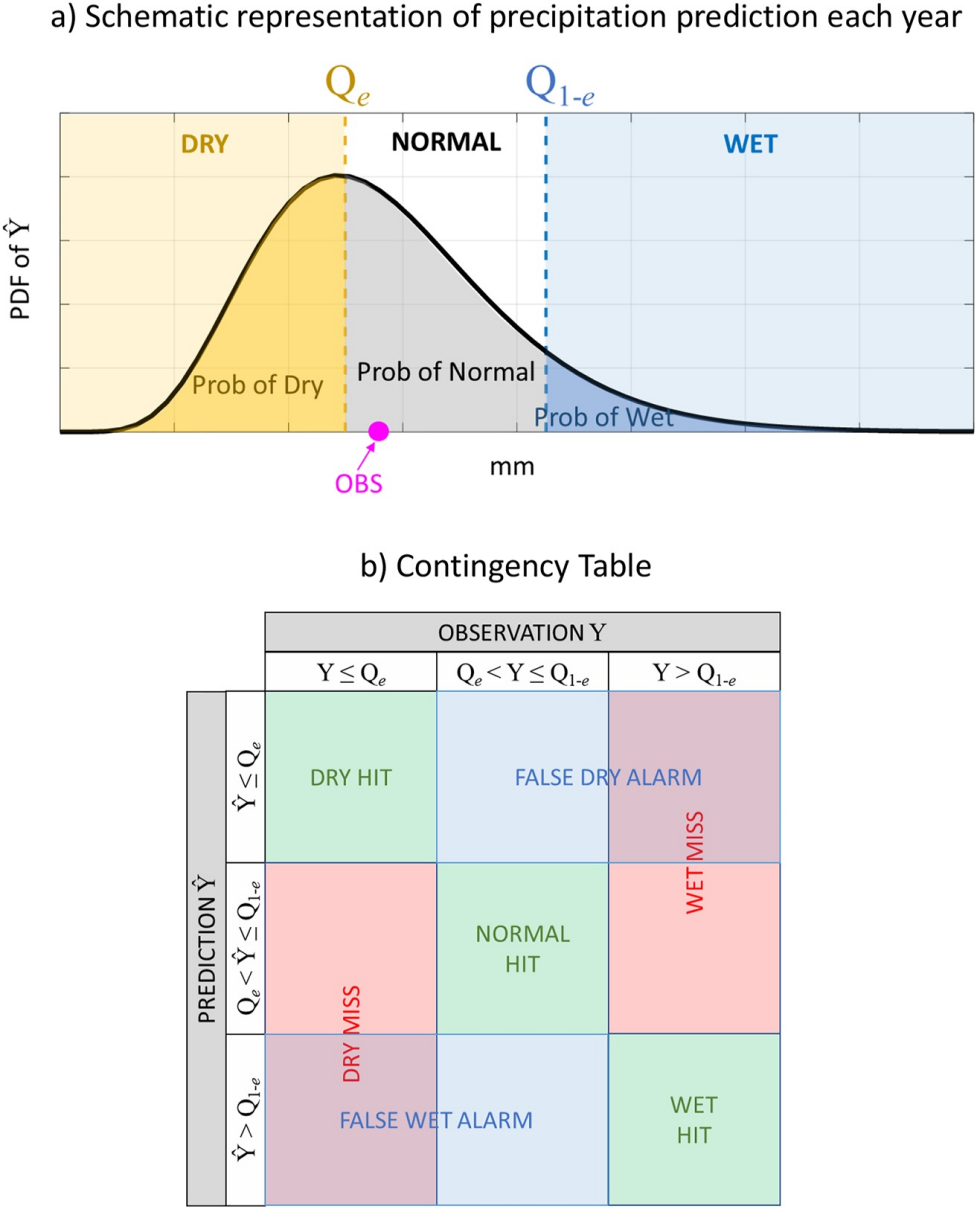
(a) Schematic of the predictive probability density function (PDF) of precipitation for each year. The prediction is made using Equation 2. (b) A 3 × 3 contingency table including all possible outcomes when predicting precipitation. Q_*e*_ denotes the *e*th quantile of the historical precipitation distribution.

For each year t=1967−1968,1968−1969, … ,2016−2017, we used SST predictors to estimate the entire predictive Probability Density Function (PDF) of precipitation; see schematic in Figure 4a. Specifically, if $f_{Y|X}^{t}$ is the conditional predictive PDF of the random variable Y (here, precipitation in Nov–Mar) in year t, given the random vector X (*M* predictors in Aug–Oct), then:

(1) $$\begin{array}{l} f_{Y|X}^{t}\left(y\right)=\frac{f_{Y,X}\left(y,\;x^{t}\right)}{f_{X}\left(x^{t}\right)}\; \end{array}$$

where $f_{Y,X}$ is the joint PDF of Y and X, and $f_{X}$ is the joint PDF of X. Since the marginal PDFs of the predictor and the predictand variables are in principle different (e.g., SST vs. precipitation), simple theoretical multivariate distributions (like the multivariate Normal or Gamma distributions) cannot be used to model the joint distributions of Y and X. To address this issue and to keep our approach simple, we used copula functions to represent the above joint distributions (see also Madadgar & Moradkhani, 2013, 2014; Madadgar et al., 2016). Equation 1 becomes (Nelsen, 1999):

(2) $$\begin{array}{l} f_{Y|X}^{t}\left(y\right)=\frac{c_{Y,X}\left(v,\;u^{t}\right)}{c_{X}\left(u^{t}\right)}\;f_{Y}\left(y\right) \end{array}$$

where $f_{Y}$ is the marginal PDF of Y, $c_{Y,X}$ and $c_{X}$ are the PDFs of the copulas, v=Pr[Y≤y] is the marginal cumulative distribution function (CDF) of Y evaluated at y, and $u^{t}={\left[u_{1}^{t},\;u_{2}^{t},\;\text{…}\;u_{i}^{t},\text{ … },u_{M}^{t}\right]}^{T}$, where $u_{i}^{t}=\text{Pr}\left[X_{i}\leq x_{i}^{t}\right]$ is the marginal CDF of the *i*th predictor $X_{i}$ evaluated at its value $x_{i}^{t}$. As previously mentioned, to fit this model we first linearly detrended all series and then fit parametric distributions to the historical precipitation totals and the indices series, to model the marginal distributions. The fitted distributions were Gamma and Gaussian, respectively, both statistically significant at *α* = 0.05, based on the Kolmogorov‐Smirnov test. To model the joint distributions, we chose to use Gaussian copulas in Equation 2, because the Gaussian copula is among the simplest models to preserve high dimensional dependencies (Joe, 1997).

To ensure that our assessment of the predictability was rigorous, we adopted a 5‐fold cross validation approach. Under this setting, the 50‐year period was separated into five, non‐overlapping and continuous 10‐year sets (i.e., the first set included the years [1967−1968,1968−1969, … ,1976−1977], the second set included the years [1977−1978,1978−1979, … ,1986−1987], etc.). For each year t in the first 10‐year set, an out‐of‐sample prediction was made by fitting the predictive model (i.e., fitting the marginal and copula distributions) to the data from the remaining 40 years. This was repeated for the four other 10‐year sets, until a single out‐of‐sample prediction was made for all 50 years. Our performance metrics (described below) were applied to the out‐of‐sample predictions. Note that the latter is a much stricter approach relative to the leave‐one‐out cross validation, which can lead to unrealistically high predictive performance (usually from overfitting), especially as the complexity of the model increases (see discussion in Section 3 and DelSole & Banerjee, 2017).

### 2.3 Statistical Metrics of Prediction Skill

In this section we describe the metrics we used to assess the predictability of precipitation across the CONUS. Predictability was assessed in each of the grid points separately, using both continuous and categorical metrics. For the continuous case, we used the coefficient of determination:

(3) $$\begin{array}{l} R^{2}=1-\frac{\sum_{t}{\left\{y_{obs}^{t}-\int_{0}^{+\infty }y\;f_{Y|X}^{t}\left(y\right)dy\right\}}^{2}}{\sum_{t}{\left\{y_{obs}^{t}\;-\;\bar{y_{obs}}\right\}}^{2}} \end{array}$$

where $\bar{y_{obs}}$ is the average observed precipitation in the corresponding grid point, and the term $\int_{0}^{+\infty }y\;f_{Y|X}^{t}\left(y\right)dy$ is equivalent to the least‐squares prediction and represents the expected value of the conditional predictive distribution $f_{Y|X}^{t}$ that comes from Equation 2. In linear settings as in our study and when predicting within sample, R^2^ is equal to the square of the Pearson correlation between the observations and the predictions and is thus a non‐negative number. However, when predicting out‐of‐sample (e.g., in a cross validation setting), overfitting the data may lead to very poor predictions (e.g., worse than a climatology‐based prediction), in which case R^2^ can be negative (see Section 3).

For the categorical case, over each grid point and for a probability of nonexceedance e, we defined historical “dry conditions” as the Nov‐Mar seasons for which the precipitation total did not exceed the e‐quantile $Q_{e}$ of the historical probability distribution. Similarly, historical “wet conditions” correspond to Nov‐Mar seasons when the precipitation total was above the historical $Q_{1-e}$. Note that as described below in our analysis, we assessed the prediction skill for several different levels of extremes by allowing e to vary.

Based on these definitions, we created a table with all possible outcomes with regard to correctly or incorrectly predicting dry, normal, or wet conditions (see Figure 4b). The latter is known as “the contingency table” (Wilks, 2006), and has been widely used in many hydrologic and weather forecasting applications, to assess the skill of correctly predicting/monitoring events in different spatiotemporal scales (Behrangi et al., 2011; Haile et al., 2012; Hao et al., 2013). A typical contingency table consists of four possible outcomes (hit, miss, false alarm, and correct rejection), which originate from predicting two possible categorical states: The occurrence or lack of occurrence of an event. In our case, we had three states (classes), that is, dry, normal, or wet totals of seasonal precipitation, thus yielding a multi‐category 3 × 3 contingency table. Multi‐category contingency tables have been discussed in the past (Brooks & Doswell, 1996; Iglesias et al., 2014; Murphy & Winkler, 1987; Zhou et al., 2011), and our study highlights their utility for identifying asymmetries in predictability of different classes (see Section 3).

Many different metrics to assess predictive skill can be defined, even when considering only two classes (AghaKouchak & Mehran, 2013; Wilks, 2006), however, the probabilities of a hit (known as probability of detection; POD) and of a false alarm (known as the false alarm ratio; FAR) are most commonly used in the literature (Anagnostou et al., 2010; Gourley et al., 2012; Hao et al., 2013). Here we used the critical success index (CSI), which combines the latter two metrics, as follows (Schaefer, 1990):

(4) $$\begin{array}{l} \text{CSI}=\frac{1}{\frac{1}{\text{POD}}\;+\;\frac{1}{1\;-\;\text{FAR}}\;-\;1}=\frac{1}{\frac{1}{\text{POD}}\;+\;\frac{1}{\text{SR}}\;-\;1} \end{array}$$

where SR=1−FAR is the success ratio. The CSI is unitless and ranges from 0 (corresponds to no prediction skill) to 1 (corresponds to perfect prediction skill). The POD and the SR of dry/normal/wet conditions or in general of a year when precipitation belongs to a set of outcomes A are, respectively:

$$\mathrm{POD}=\mathrm{Pr}\left[\hat{Y}\in A\;|\;Y\in A\right]$$

(5) $$\begin{array}{l} \text{SR}=\mathrm{Pr}\left[Y\in A\;|\;\hat{Y}\in A\right]=\frac{\text{POD}\;*\;\mathrm{Pr}\left[Y\;\in \;A\right]}{\mathrm{Pr}\left[\hat{Y}\;\in \;A\right]} \end{array}$$

where Y is the observed precipitation value, $\hat{Y}$ is the prediction, and the last equation arises from Bayes' theorem. In other words, in both metrics one considers the number of correct predictions or hits of an event (e.g., dry conditions), and divides it by the number of total observed events in POD and by the number of total predicted events in SR (Schaefer, 1990).

When incorporating the uncertainty of the prediction (as in the present study), the POD and SR cannot be trivially calculated as described above, but rather need to be based on the integration of the probability distribution between thresholds that define different precipitation classes (e.g., the quantiles $Q_{e}$ and $Q_{1-e}$). Specifically, by inserting Equation 5 into 4, we derived the modified dry, wet and normal CSI metrics to assess the predictability of dry, wet and normal conditions, respectively. We examined different values of *e* (i.e., 0.25, 0.333), and for each case, we calculated the values of the metrics in the period from 1967–1968 to 2016–17.

(6) $$\begin{array}{l} \text{Dry}\;\text{CSI}= \end{array}{\left[\frac{1}{\mathrm{Pr}\left[\hat{Y}\;\leq \;Q_{e}\;|\;Y\;\leq \;Q_{e}\right]\;}\;+\;\frac{1}{\frac{\mathrm{Pr}\left[\hat{Y}\;\leq \;Q_{e}\;|\;Y\;\leq \;Q_{e}\right]\;\mathrm{Pr}\left[Y\;\leq \;Q_{e}\right]}{\mathrm{Pr}\left[\hat{Y}\;\leq \;Q_{e}\right]}}-1\right]}^{‐1}$$

The term $\mathrm{Pr}\left[\hat{Y}\leq Q_{e}\;|\;Y\leq Q_{e}\right]$ corresponds to the probability of the predictive model detecting an actual drought (i.e., dry POD), and it can be easily calculated as the integral $\int_{0}^{Q_{e}}f_{Y|X}^{\text{Dry}}\left(y\right)dy$, where $f_{Y|X}^{Dry}$ is the composite PDF of all predictive PDFs during the actual dry Nov‐Mar seasons within the period from 1967–1968 to 2016–2017. The term $\mathrm{Pr}\left[\hat{Y}\leq Q_{e}\right]$ represents how often the predictive model predicts dry conditions. Lastly, note that the term $\mathrm{Pr}\left[Y\leq Q_{e}\right]$ is by definition equal to *e*.

We similarly defined:

(7) $$\begin{array}{l} \text{Wet}\;\text{CSI}={\left[\frac{1}{\mathrm{Pr}\left[\hat{Y}\;>\;Q_{1-e}\;|\;Y\;>\;Q_{1-e}\right]\;}+\frac{1}{\frac{\mathrm{Pr}\left[\hat{Y}\;>\;Q_{1-e}\;|\;Y\;>\;Q_{1-e}\right]\;\mathrm{Pr}\left[Y\;>\;Q_{1-e}\right]}{\mathrm{Pr}\left[\hat{Y}\;>\;Q_{1-e}\right]}}-1\right]}^{-1} \end{array}$$

(8) $$\begin{array}{l} \text{Normal}\;\text{CSI}={\left[\frac{1}{\mathrm{Pr}\left[Q_{e}\;<\;\hat{Y}\;\leq \;Q_{1-e}\;|\;Q_{e}\;<\;Y\;\leq \;Q_{1-e}\right]\;}+\frac{1}{\frac{\mathrm{Pr}\left[Q_{e}\;<\;\hat{Y}\;\leq \;Q_{1-e}\;|\;Q_{e}\;<\;Y\;\leq \;Q_{1-e}\right]\;\mathrm{Pr}\left[Q_{e}\;<\;Y\;\leq \;Q_{1-e}\right]}{\mathrm{Pr}\left[Q_{e}\;<\;\hat{Y}\;\leq \;Q_{1-e}\right]}}-1\right]}^{-1} \end{array}$$

Apart from the CSI for each of the different classes of precipitation (dry, normal, wet), we also introduced a summarizing statistical metric which considers the skill with regard to all classes in combination. This statistic represents, in logarithmic scale, the probability assigned by the considered model to the correct (observed) precipitation class per year, that is, the model‐based likelihood of the observed class per year. The categorical log‐likelihood of the data is defined as:

(9) $$\begin{array}{l} \text{Log}L=\;\frac{1}{N}\text{Log}\left[\prod_{t}\left(I_{D}^{t}\int_{0}^{Q_{e}}f_{Y|X}^{t}\left(y\right)dy+I_{N}^{t}\int_{Q_{e}}^{Q_{1-e}}f_{Y|X}^{t}\left(y\right)dy+I_{W}^{t}\int_{Q_{1-e}}^{+\infty }f_{Y|X}^{t}\left(y\right)dy\right)\right]\; \end{array}$$

where N is the number of years (in our case N=50). The three integrals in Equation 9 represent the probability of dry, normal, and wet conditions in year t, as estimated from the model given $x^{t}$. For any t, the coefficients (indicator functions) of the integrals indicate whether the year was actually dry, normal, or wet:

$$I_{D}^{t}=\left\{\begin{array}{l} 1,\;0\leq y_{\mathrm{obs}}^{t}\leq Q_{e}\\ 0,\;\text{otherwise} \end{array}\;\right.,\;I_{N}^{t}=\left\{\begin{array}{l} 1,\;Q_{e}<y_{\mathrm{obs}}^{t}\leq Q_{1-e}\\ 0,\;\text{otherwise} \end{array}\;\right.,\;I_{W}^{t}=\left\{\begin{array}{l} 1,\;y_{\mathrm{obs}}^{t}>Q_{1-e}\\ 0,\;\text{otherwise} \end{array}\;\right.$$

To be able to assess statistical significance when using the above metrics, we defined the null hypothesis, H_0_: *“The predictand* Y *is independent of the predictors* X *, and thus,* X *are non‐informative for prediction”*.

Under H_0_, the copula ratio in Equation 2 is equal to unity and the predictive distribution of precipitation for any t and for any grid point is equal to the historical marginal PDF of precipitation, $f_{Y|X}^{t}\left(y\right)=f_{Y}\left(y\right)$; that is, predictions under H_0_ are based on the “baseline climatology” of Y. As such, the asymptotic (N→∞) values of the considered statistical metrics under H_0_, can be easily derived:

$$R_{H_{0}}^{2}=0$$

$${\text{Dry}\;\text{CSI}}_{H_{0}}=\frac{e}{2-e}$$

$${\text{Wet}\;\text{CSI}}_{H_{0}}=\frac{e}{2-e}$$

$${\text{Normal}\;\text{CSI}}_{H_{0}}=\frac{1-2e}{1+2e}$$

$$\text{Log}L_{H_{0}}=2e\text{Log}\left[e\right]+\left(1-2e\right)\text{Log}\left[1-2e\right]$$

However, because of the limited sample size, N=50, the asymptotic values cannot be used to assess significance, that is, to reject the H_0_. In other words, for a predictive model with no true predictive skill, the value of a metric may randomly result in a value that is higher than its asymptotic value. Thus, we used the *F* statistic for the R^2^ and Monte Carlo simulations to obtain the critical values of the rest of the statistics, for a significance level of *α* = 0.05. Particularly, we simulated 5,000 pairs of independent Y and X series (of sample size N=50), and determined the empirical 95%‐quantile for each of the above categorical statistics and for different definitions of the extremes (*e* values), which we then used to assess significance.

## 3 Results

**Figure 5:**
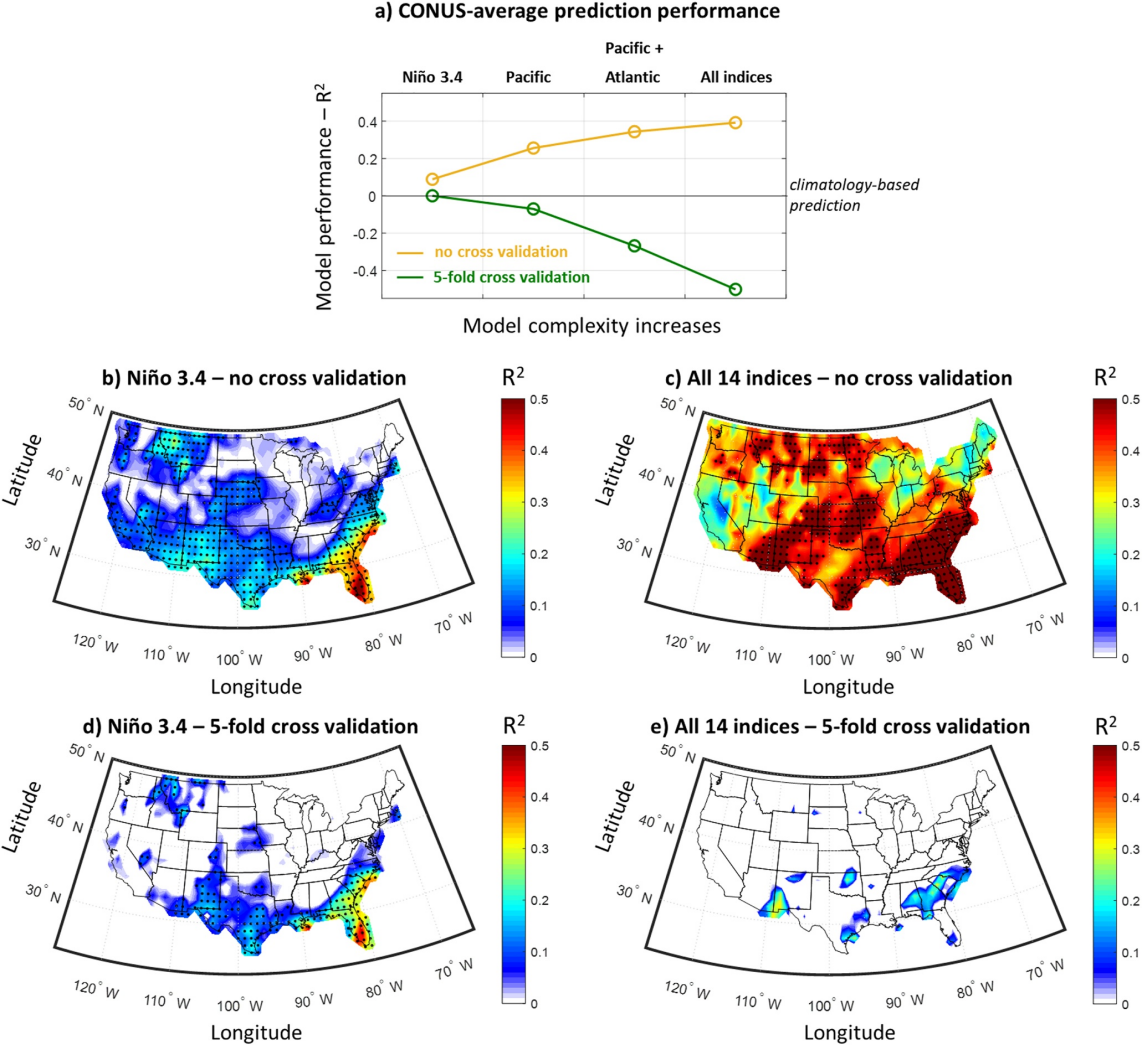
Performance of seasonal prediction models for contiguous United States (CONUS) precipitation using the 14 SST indices. (a) Coefficient of determination (R^2^) of the prediction mean for increasingly complex predictive models, with and without applying a 5‐fold cross validation technique. The CONUS‐average R^2^ is presented. (b) Coefficient of determination of the prediction mean across CONUS when using the Niño 3.4 index, and without applying a 5‐fold cross validation technique. Stippling indicates statistical significance (*p* < 0.05) as assessed using the *F* statistic. (c) Same as in (b), but when using all 14 SST indices. (d and e) Same as in (b and c), but when applying a 5‐fold cross validation technique. Results in this figure clearly show the overestimation of the predictive skill when not applying a cross validation technique, and the drastic effect of overfitting when the model complexity increases.

We start by assessing the predictive skill when using the 14 SST indices to predict precipitation across the CONUS and under two settings: When no cross validation is performed and when a 5‐fold cross validation is performed (Figure 5). For our assessment, we used the coefficient of determination (R^2^) between the mean of the prediction and the observations. Our results show that when no cross validation is performed, adding SST indices in the predictive model and increasing model complexity leads to a much better predictive skill (Figures 5a–5c). In fact, when using all 14 indices, precipitation predictability seems very high over the entire CONUS, with a CONUS‐average R^2^ on the order of 0.4 (i.e., the predictive model explains 40% of precipitation variability, on average). However, when applying a 5‐fold cross validation technique that resembles an out‐of‐sample prediction setting, predictive performance is much lower, highlighting the effect of overfitting (Figures 5a, 5d and 5e). Even when using a single SST index for prediction (e.g., the Niño 3.4 index), the cross validation technique leads to lower estimate of predictability (compare panels d and b), which highlights the importance of applying cross validation techniques to avoid overestimating predictability (see also DelSole & Banerjee, 2017). Thus, for the remainder of the study we only present results where the 5‐fold cross validation has been implemented. In the cross validation case and when only using the Niño 3.4 index, one can see some regions mainly in the Southwest and Southeast where there is significant predictive skill, specifically reaching almost *R* ^2^ = 0.5 over Florida (Figure 5d). When adding all Pacific indices, the predictive skill slightly decreases (Figure 5a), and continues to decrease when all 14 indices are used for prediction (Figure 5e), illustrating the increasing effect of overfitting that occurs when more predictors are added into the model.

**Figure 6:**
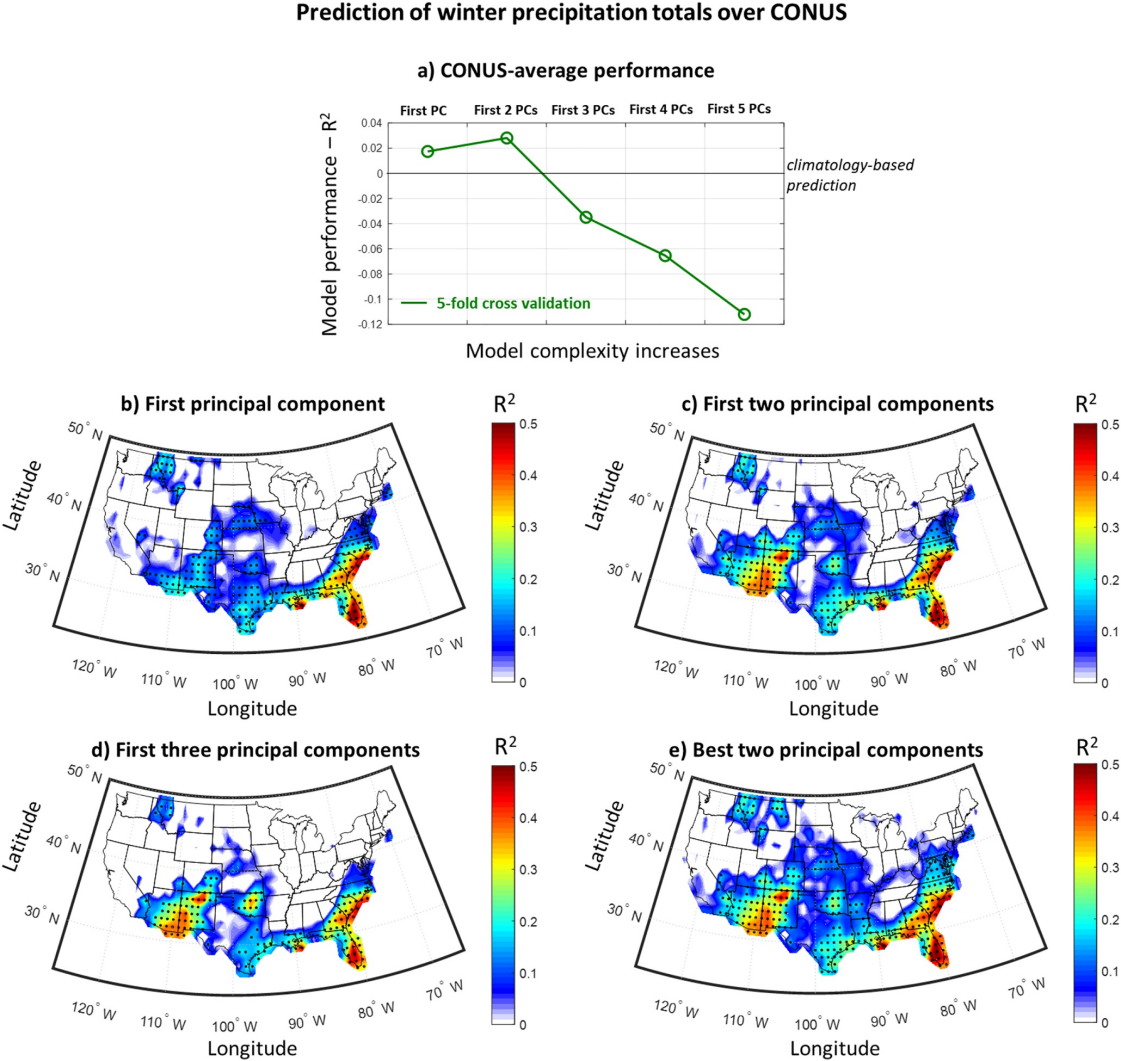
Performance of seasonal prediction models using the PCs of the climate indices. (a) Coefficient of determination (R^2^) of the prediction mean for increasingly complex predictive models, using the indices' principal components (PCs) and applying a 5‐fold cross validation technique. The CONUS‐average R^2^ is presented. (b) R^2^ of the prediction mean across contiguous United States (CONUS) when using the first PC as a predictor. Stippling indicates statistical significance (*p* < 0.05) as assessed using the *F* statistic. (c) Same as in (b), but when using the first two PCs as predictors. (d) Same as in (b), but when using the first three PCs as predictors. (e) Same as in (b), but when using all possible combinations of two predictors selected from the first five PCs and presenting the performance of the suite of models that lead to the best prediction. Results in this figure highlight predictability hotspots over the Southwest (Arizona, New Mexico), the Southeast (Florida, Georgia, South and North Carolina, Virginia), the South (u) and the northern Rockies (Idaho, Montana).

To limit the effect of overfitting, we subsequently examined the use of PCs of the SST indices as predictors of precipitation. Since the PCs are compressed representations of the information contained in the SST indices, their use allowed us to retain most of the SST information, without considerably increasing the model complexity. Our results show that the predictive skill slightly increases when using the first two PCs instead of only the first PC (see Figure 6 panel a, and compare panels b and c) and it decreases when using the first three PCs (panel d). When we added more components as predictors, the predictive skill continued to decrease (panel a). This result implies that for our study design examining seasonal precipitation predictability using a sample size of 50 years, models with more than two predictors appear prone to overfitting. To investigate the upper limit of prediction skill, we considered all possible combinations of predictive models using two out of the first five PCs (i.e., 5 × 4/2 = 10 combinations). In Figure 6e, we present the R^2^ of the prediction for the best performing combination, for each grid point separately. This figure shows that predictive skill increases slightly in some regions compared to panel (c), including western Texas, Arkansas and Montana.

Independent of the predictive model, Figure 6 shows that there are several coherent regions where precipitation is most predictable. Hotspots with relatively high levels of predictability are (a) Arizona and New Mexico in the Southwest with R^2^ ranging from 0.2 to 0.4 and (b) Florida, Georgia, South Carolina, North Carolina and Virginia in the Southeast with R^2^ ranging from 0.2 to 0.5. Hotspots with moderate levels of predictability include (a) Texas, Louisiana, and Oklahoma across the South with R^2^ mostly on the order of 0.2, and (b) northern Idaho and western Montana in the Northern Rockies with R^2^ on the order of 0.2. With our approach, we were unable to identify high levels of predictability for most of the Northeast and Midwest and for coastal states in the West.

**Figure 7:**
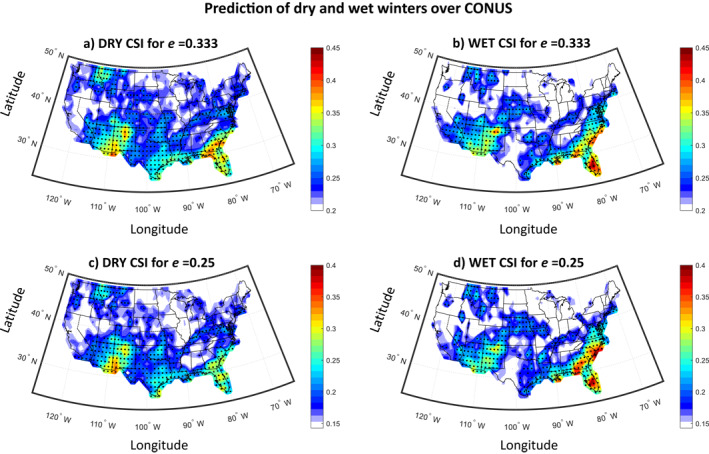
Predictability of dry and wet winters using the suite of predictive models in Figure 6e (a) Dry Critical Success Index (CSI) across contiguous United States (CONUS) for *e* = 0.333 (the dry and wet conditions correspond to the lowest and highest 1/3 of the distribution). (b) Same as in (a), but predicting wet winters. (c and d) Same as in (a and b), but for *e* = 0.25 (the dry and wet conditions correspond to the lowest and highest 1/4 of the distribution). In all panels, stippling indicates statistical significance (*p* < 0.05).

To better understand the predictability of extremes, we then examined the skill in predicting the occurrence of dry, normal and wet conditions, using the models that combine the best two PCs in each grid point (i.e., the suite of predictive models shown in Figure 6e). We present the dry and wet CSI for different values of *e* (0.25 or 0.333, that is, dry and wet conditions correspond to the lowest and highest 1/4 or 1/3 of the historical distribution) across the CONUS in Figure 7. Our results indicate that over the hotspots of predictability identified above, both dry and wet conditions are significantly predictable, independent of the value of *e*. Particularly, in the Southwest and Southeast, the prediction exhibits a CSI on the order of 30%–35%, for *e* = 0.333 (assessed as statistically significant; *p* < 0.05). Over the Northern Rockies region, the CSI is on the order of 30%. Moreover, considering the full spatial pattern of predictability shown in Figure 7, results indicate that there are some regions where the predictability exhibits an asymmetry, that is, wet conditions are more predictable than dry or vice versa. For example, wet conditions seem to be more predictable relative to dry conditions over the Southeast (most notably in Florida) and in Nebraska, while dry conditions are relatively more predictable over western Texas, Oklahoma, and Idaho.

**Figure 8:**
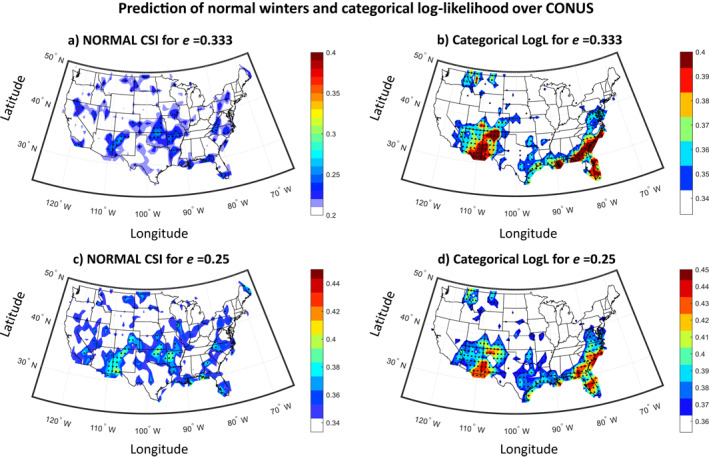
Predictability of normal winters and categorical log‐likelihood using the suite of predictive models in Figure 6e (a) Normal Critical Success Index (CSI) across contiguous United States (CONUS) for *e* = 0.333 (the dry and wet conditions correspond to the lowest and highest 1/3 of the distribution). (b) Same as in (a), but the categorical log‐likelihood across CONUS is presented. (c and d) Same as in (a and b), but for *e* = 0.25 (the dry and wet conditions correspond to the lowest and highest 1/4 of the distribution). In all panels, stippling indicates statistical significance (*p* < 0.05).

Analysis of the predictability of normal conditions (Figure 8) shows that normal conditions are much less predictable than dry/wet conditions, and that the prediction skill is for most regions statistically indistinguishable from the skill that corresponds to a climatology‐based prediction (see results considering either value for *e* in Figures 8a and 8c). Intuitively, this result of virtually no predictability for normal conditions is consistent with the concept of forecasts of opportunity, which asserts that there are time windows of little or no predictability, when large‐scale climate oscillations (i.e., potential drivers of regional hydroclimate) are inactive. On the contrary, when the climate oscillations become active (e.g., with ENSO moving into either a strong El Niño or La Niña phase), atmospheric forcing and teleconnections may be stronger, providing a more robust signal to mid‐latitude precipitation that is less likely to be masked by internal noise, which, in turn, may enhance predictability on seasonal timescales for precipitation extremes (see also Mayer & Barnes, 2021). The maps of the categorical log‐likelihood in Figures 8b and 8d clearly highlight the hotspots of predictability that were identified earlier. On average, results show that predictive models assign a probability of 0.35–0.4 to the correct class of precipitation over these regions. Note that despite being statistically significant, the latter predictive skill is limited, thus, new methods for improving prediction are warranted (see the discussion in the concluding section).

**Figure 9:**
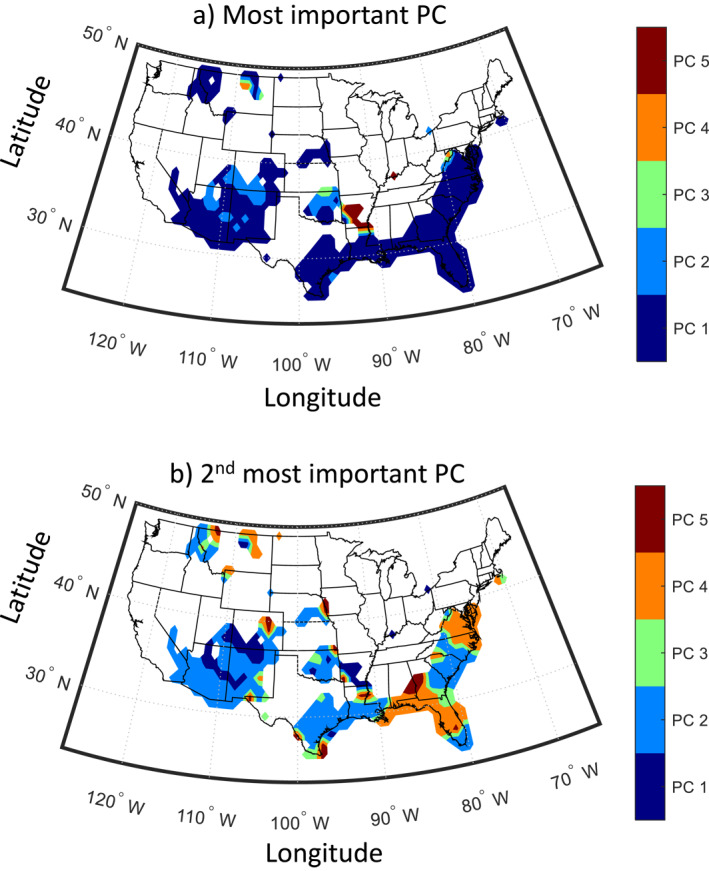
Investigation of sources of predictability. (a) Map of the most important PC (corresponding to the highest regression coefficient) used for prediction in Figure 6e (b) Same as in (a), but for the second most important principle component (PC). In both panels, we present results only for points where the value of R^2^ in Figure 6e is statistically significant (*p* < 0.05).

In a final step, we explored the sources of predictability. Specifically, for each grid point, we created a bivariate linear regression model using the two predictors identified in Figure 6e. We present the PCs that received the highest (lowest) regression coefficient in terms of absolute values in Figure 9a (Figure 9b). Our results show that on average PC1 and PC2 are contributing the most to predictability. This means that precipitation variability across the CONUS is linked with SST over the Pacific basin (PC1) and over the North Atlantic basin (PC2), which is consistent with past literature, for example, see Dai (2013); Enfield et al. (2001); McCabe et al. (2004); Newman et al. (2016). Specifically, over all predictability hotspots that were identified herein, Pacific Ocean SST is the most important predictor of precipitation. Over the Southwest, the Atlantic Ocean SST is also an important contributor to precipitation predictability; we find that the regression coefficients of PC1 and PC2 are almost of the same order. Over, the Northern Rockies and the Southeast, the second most important contributor is PC4 (Figure 9b), although the regression coefficient with this PC is relatively small (not shown) in both regions. This means that over these regions, the Pacific SST is the main source of predictability.

## 4 Discussion and Concluding Remarks

In this study, we present a rigorous probabilistic framework for assessing precipitation (or other climate variables) predictability under uncertainty. Our specific aim has been to gain insight about hotspots of predictability, which are defined as regions where precipitation is inherently more predictable than in surrounding regions. Our framework uses copula modeling to estimate the entire predictive distribution and quantify uncertainty, and principal component analysis and a cross validation technique to reduce dimensionality and avoid the impact of overfitting. In our analysis, we also evaluate the predictability of dry, normal and wet conditions using a multi‐category 3 × 3 contingency table, a modified critical success index, and a categorical loglikelihood evaluation metric.

We applied our framework focusing on the problem of seasonal prediction of precipitation over CONUS. We used SST information that is averaged during late boreal summer and early fall (Aug–Oct) to assess predictability of CONUS precipitation during the following Nov–Mar – a time of the year with elevated fire risk for southeastern states and when most precipitation occurs over the western states. Our results indicate that Nov‐Mar precipitation totals are most predictable for several states in the Southwest, including Arizona and New Mexico, and across several states in the Southeast, including Florida, Georgia, South Carolina, North Carolina, and Virginia. Additional hotspots with moderate levels of predictability occur in the Northern Rockies and in southern states, including Texas, Oklahoma, and Louisiana. We find that SST information can be used to predict the occurrence of dry and wet conditions, while normal conditions are more difficult to predict. This result illustrated the advantage of using multi‐category contingency tables (not usually employed in the seasonal prediction literature) for identifying asymmetries in predictability of different states. We also establish that the biggest source of precipitation predictability during the last 50 years has been the SST over the Pacific and north Atlantic Oceans. The identified hotspots as well as sources of predictability align reasonably well with previous reports. Specifically, many past studies have identified statistically significant skill in prediction of precipitation totals for the Southwest (Gibson et al., 2021; Liu et al., 2018; Madadgar et al., 2016; Mamalakis et al., 2018; McCabe & Dettinger, 1999; Pan et al., 2019; Schonher & Nicholson, 1989; Stevens et al., 2021; Zhang et al., 2018 among many others), for Gulf Coast and Southeast regions (Becker et al., 2014; Devineni & Sankarasubramanian, 2010a, 2010b; Kirtman et al., 2014), and for precipitation extremes in the Northwest (Gershunov & Cayan, 2003; Zarekarizi et al., 2018). Moreover, previous studies have highlighted the Pacific and North Atlantic Ocean basins as the main sources of precipitation predictability over North America (see e.g., Dai, 2013; Enfield et al., 2001; McCabe et al., 2004; Newman et al., 2016).

Our results defining the spatial pattern of precipitation predictability may help improve water resources management in the US (as well as other regions; Delorit & Block, 2019; Giuliani et al., 2020), by informing the design of operational systems. Moreover, the probabilistic framework introduced herein can be applied not only to historical data (retrospectively), but also to climate model outputs in order to explore possible future changes in the strength of climate teleconnections and predictability, which are important for assessment of climate change impacts on regional hydroclimate.

Future extensions of our framework may explore using different marginal distributional models or different copula models (other than the Gaussian) that exhibit tail dependence. It is possible that previously reported asymmetries in teleconnections to the US hydroclimate (Feng et al., 2017; Zhang et al., 2014) are more efficiently modeled with non‐Gaussian copulas. Second, although in this study we aimed to limit the effect of non‐stationarity by detrending and focusing on the most recent 50 years, non‐stationary dependencies and their effect on statistically based predictive skill cannot be totally ruled out. Thus, future work should focus on developing ways to incorporate changes in for example, ENSO teleconnections in a warming climate (Beverley et al., 2021; Fasullo et al., 2018; Perry et al., 2017; Sun et al., 2020; Yan et al., 2020). Last, it may be relatively straightforward to extend our approach to assess predictability of other aspects of the precipitation distribution, even beyond the ones considered here, for example, by defining extremes based on specified magnitudes (instead of quantiles), and/or by using additional statistical metrics, important for risk quantification and water management decisions.

We conclude by highlighting that our results show that predictability is low over many areas on the CONUS west coast (i.e., Washington, Oregon and northern California), for which Nov‐Mar is the season when the majority of annual precipitation occurs (see Figure 2a). This low predictability is a consequence of the weak relationship between the predefined SST indices and western US precipitation, and has also been confirmed in work using dynamical models (see Becker et al., 2014; Kirtman et al., 2014). This low predictability places important constraints on decision making, mitigation of hazards and water resources management. Toward improving predictive skill, future work could use data‐driven (going beyond predefined indices) machine learning methods that are designed to account for high dimensionality and spatiotemporal dependencies of predictor variables. Such methods have shown considerable potential (e.g., see data‐driven and machine/deep learning methods in DelSole & Banerjee, 2017; Liu et al., 2018; Giuliani et al., 2019; Ham et al., 2019; Stevens et al., 2021; Gibson et al., 2021; Peng et al., 2021 among others), and are suited for investigating seasonal precipitation predictability in nonlinear settings. Further improvements may be possible by combining statistical and dynamical model predictions in a hybrid, post processing setting (Hao et al., 2018; Khajehei et al., 2018; Khajehei & Moradkhani, 2017; Madadgar et al., 2016).

## Conflict of Interest

The authors declare no conflicts of interest relevant to this study.

## Data Availability

The data used in our analysis are all freely available online. Precipitation data over CONUS can be found at https://psl.noaa.gov/data/gridded/data.unified.daily.conus.html. SST data are downloaded from https://www.esrl.noaa.gov/psd/data/gridded/data.cobe2.html, while PDO series can be found at https://www.esrl.noaa.gov/psd/data/climateindices/list. The code that was used for this work can be found at: https://doi.org/10.5281/zenodo.6316579.
